# *Candida parapsilosis* Infection After Crescentic Lamellar Wedge Resection in Pellucid Marginal Degeneration

**DOI:** 10.4274/tjo.23865

**Published:** 2018-06-28

**Authors:** Selma Özbek-Uzman, Ayşe Burcu, Züleyha Yalnız-Akkaya, Evin Şingar-Özdemir, Firdevs Örnek

**Affiliations:** 1 University of Health Sciences, Ankara Training and Research Hospital, Ophthalmology Clinic, Ankara, Turkey

**Keywords:** Pellucid marginal degeneration, crescentic lamellar wedge resection, Candida parapsilosis, fungal keratitis, iatrogenic trauma

## Abstract

Infectious keratitis after corneal lamellar surgery is a rare complication. In this report, we present unexpected complications after crescentic lamellar wedge resection (CLWR) and their treatment in a patient with pellucid marginal degeneration. A 42-year-old male patient developed fungal keratitis due to *Candida parapsilosis* in the late postoperative period after CLWR. Infection was controlled with medical treatment. However, recurrent intraocular infections and cataract formation occurred, probably due to capsular damage and inoculation of microorganisms into the crystalline lens during antifungal drug injection. Lensectomy was performed due to cataract progression and recurrence of the infection when treatment was discontinued. Amphotericin B was administered to the anterior chamber at the end of the operation. Four months later, an intraocular lens was implanted and corneal cross-linking treatment was performed. At the last visit, visual acuity reached 9/10. This case shows that good visual acuity can be achieved with appropriate treatment of fungal keratitis and all associated complications after CLWR.

## Introduction

Pellucid marginal degeneration (PMD) is a bilateral, asymmetric, noninflammatory ectatic disorder of the cornea. Cornea thinning typically occurs in a 1-2 mm band parallel to the limbus between 4 and 8 o’clock.^1,2^

Glasses and contact lenses are sufficient for visual rehabilitation in the early stages but surgical treatment is necessary in advanced stages. In crescentic lamellar wedge resection (CLWR), the abnormally thin corneal stroma is removed while sparing the central cornea and the margins of normal-thickness stroma are reapposed.^3,4,5,6^

Infectious keratitis after keratoplasty procedures is a rare but serious complication. The incidence is reported as 1.5-12.6% after full-thickness techniques.^7,8^ There are few publications in the literature regarding lamellar surgeries.^7,8,9^ There are no previous reports of keratitis after CLWR for PMD.

In this article, we present a case of unilateral *Candida parapsilosis* infection after bilateral CLWR for PMD and the unexpected complications that occurred during its treatment.

## Case Report

**Figure 1:**
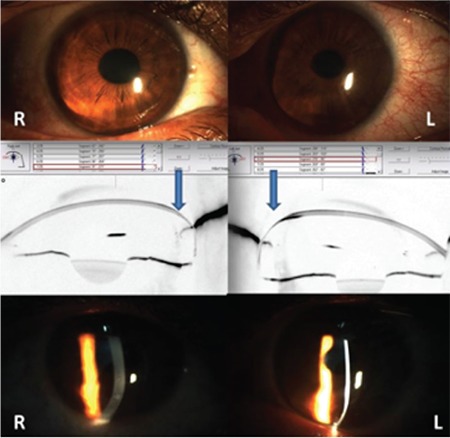
Preoperatively, both eyes show inferior corneal steepening and stromal thinning, while perilimbal stromal thickness is normal R: Right eye, L: Left eye

**Figure 2:**
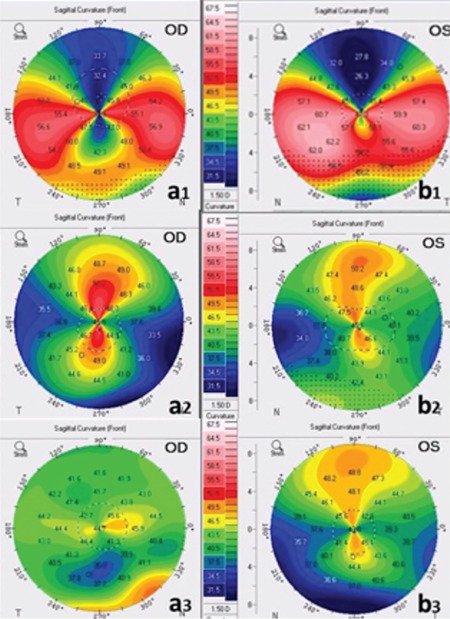
a1,b1) Topography in initial examination revealed typical crab claw pattern and topographic astigmatism was 21.2 dioptri (D) in the right eye and 23.8 D in the left eye; a2,b2) At postoperative day 15, astigmatism was markedly reduced at 15.3 D and 9.8 D in the right and left eyes, respectively; a3,b3) At postoperative 23 months, astigmatism was 6.1 D in the right and 1.4 D in the left eye OD: Right eye, OS: Left eye

A 42-year-old male refugee under follow-up for PMD had an uncorrected visual acuity (UCVA) in the right eye of counting fingers from 4 m and best corrected visual acuity (BCVA) of 2/10 with refraction values of -5.00, -12.00 α 35, topographic astigmatism (TA) of 21.2 dioptri (D) α 95. In the left eye, UCVA was counting fingers from 2 m, BCVA was 1/10 with refraction of -6.00, -14.00 α 45 and TA of 23.8 D α 93.5 (Figure 1, Figure 2 a1-b1). Bilateral CLWR was planned for both eyes due to insufficient visual improvement with spectacles and contact lens incompatibility.

The borders of the area to be excised were mapped onto the cornea preoperatively under the biomicroscope light using a 27-gauge needle. Under general anesthesia, a crescent blade was used to make a crescent-shaped incision in the cornea including the area of thinning between 4-8 o’clock, 1-2 mm from the limbus. Stromal dissection from the incision to just above the Descemet’s membrane was done and the thinned corneal stroma was resected using a crescent blade and scissors. After ensuring the Descemet’s membrane was intact, the upper and lower normal-thickness corneal tissue was reapposed using five 10/0 sutures, followed by paracentesis through the limbus to reduce intraocular pressure. The five previously placed sutures were knotted and eight 10/0 polypropylene sutures were added. Topical antibiotic (0.5% moxifloxacin, 4 times daily), topical corticosteroid (1% prednisolone acetate, 4 times daily) and artificial tear drops were prescribed postoperatively. Topography was performed at each postoperative visit. Loose sutures were replaced. The same surgical procedure was performed in the right eye 3 months after the left eye.

On postoperative day 15, UCVA was 5/10, BCVA of 7/10 with refraction of +1,00, -4.50 α 55 and TA was 15.3 D α 167 in the right eye and UCVA was 6/10, BCVA was 9/10 with refraction of +2.00, -4.00 α 70 and TA was 9.4 D α 10 in the left eye (Figure 2 a2-b2).

**Figure 3:**
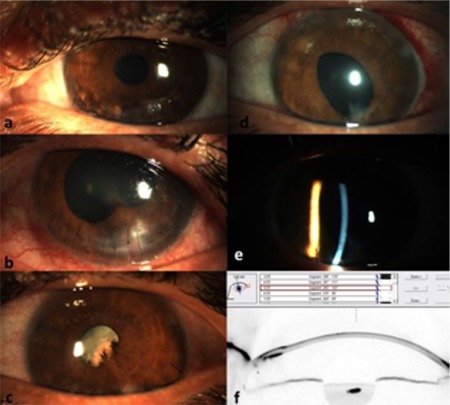
Images of the left eye. a) At 5 months, a single slack suture and a keratitis focus at 5 o’clock; b) At 7 months, localized lens opacity and posterior synechia; c) Cataract progression and capillaries extending from the iris margin onto the lens; d,e) pseudophakia; f) Scheimpflug section after corneal cross-linking

Slit-lamp examination at postoperative 5 months revealed a single loose suture at 5 o’clock on the resection line in the left eye, a 1x2 mm area of stromal infiltrate, mild edema surrounding the infiltrate and inflammatory reaction in the anterior chamber (+2 cells) (Figure 3a). After taking samples for direct microscopy and culture, treatment with topical fortified vancomycin 50 mg/mL 8 times daily, ceftazidime 50 mg/mL 8 times daily and 2% fluconazole 6 times daily was initiated. Direct microscopy of corneal scraping showed yeast and culture produced *Candida parapsilosis*. Antibiogram results indicated sensitivity to fluconazole, voriconazole and amphotericin B. Based on these findings, the fortified vancomycin and ceftazidime were discontinued and treatment was continued with 2% fluconazole drops hourly and oral fluconazole 200 mg daily. Initial response to this therapy was good. However, after 5 weeks the patient exhibited enlargement of the lesion, extensive keratic precipitates throughout the cornea and hypopyon in the anterior chamber. UCVA was 2/10 and fundus examination and ultrasonography revealed no signs of endophthalmitis. Suspecting resistance to the antifungal therapy, the agent was changed to topical 0.15% amphotericin B (amph B) hourly. After taking a sample from the anterior chamber under local anesthesia, 3 injections of 7.5 µg/0.1 mL amph B were administered at 72-hour intervals. Four days after the procedure, the hypopyon disappeared, the anterior chamber reaction regressed and the lesion was diminished in size. However, after the third injection, the patient developed hyphema nearly filling the anterior chamber. The hyphema regressed on day 7, revealing lens opacification and posterior synechia at 5 o’clock, just opposite the incision (Figure 3b). During follow-up, the patient experienced three infectious episodes with hypopyon at intervals of five to seven weeks after discontinuing antifungal therapy. Infection was controlled by resuming antifungal therapy. Lensectomy and synechotomy were performed without intraocular lens implantation while the patient continued antifungal therapy due to cataract progression and recurrence of the infection after treatment was discontinued (Figure 3c). At the end of the procedure, 7.5 µg/0.1 mL amph B was administered to the anterior chamber and topical antifungal therapy was continued for another month. Four months after cataract surgery, an intraocular lens was implanted in the sulcus in a second procedure (Figure 3d). Two months later, corneal stability was achieved in the left eye by performing corneal cross-linking therapy (Figures 3e-3f).

**Figure 4:**
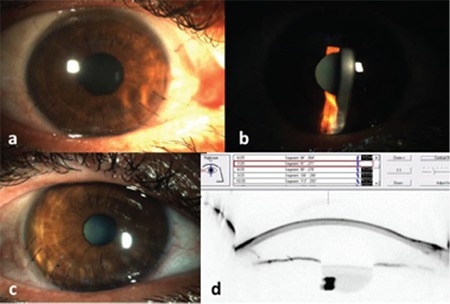
Postoperative images of the right eye, a,b) day 15; c) 21 months; d) Scheimpflug section

There were no intraoperative or early postoperative complications in the right eye (Figure 4).

At 23 months after the first operation, the right eye had BCVA of 8/10 with refraction of -1.00, +2.00 α 135 and TA of 6.1 D α 104, while the left eye had BCVA of 9/10 with refraction of +1.50, -4.00 α 65 and TA of 1.4 D α 50 (Figure 2 a3-b3).

## Discussion

PMD typically usually shows bilateral involvement of the inferior cornea 1-2 mm from the limbus. Surgical treatment is difficult due to the peripheral location of the ectasia.^5,6^ Some surgical techniques that can be used include large-diameter penetrating keratoplasty (PK), crescentic lamellar keratoplasty, crescentic lamellar keratoplasty combined with PK, CLWR, tuck-in keratoplasty, lower-quadrant eccentric PK and corneoscleroplasty.^3,4,5,6,10,11^

In CLWR, a narrow crescent of peripheral tissue is excised to remove the thinned corneal stroma and reduce astigmatism.^11^ Advantages of this technique are that the normal central cornea is preserved and there is no risk of graft rejection, primary graft failure, or interface haze because donor tissue is not used. As steroids are used for a shorter time, there is also low risk of developing steroid-related complications. The deeper corneal layers remain intact, thus providing a stronger incision site and shorter visual recovery time. In addition, risk of retinal detachment, choroidal detachment and endophthalmitis is low because the only invasive procedure performed to the anterior chamber is a small paracentesis.^2^ CLWR was performed in our patient to avoid graft-related complications and provide rapid visual rehabilitation. There was significant early visual improvement in both eyes and excellent outcomes were achieved at 2-year follow-up. At 23 months after resection, TA decreased to 21.2 D to 6.1 D in the right eye and 23.8 D to 1.4 D in the left eye.

There are many predisposing factors for the development of keratitis after corneal surgeries. Suture-related problems (43-60%), persistent epithelial defects (38-74%), topical medication use (40-81%), low socioeconomic status (60%), soft contact lens use (9-45%) and lid anomalies (23%) are the most commonly reported.^7,8,12,13^ In developed countries, *Candida albicans* is the most frequently isolated fungus in corneal infections; however, the prevalence of *Candida parapsilosis *is increasing.^9,14^ New keratoplasty techniques may reduce the rate of postoperative infectious keratitis but retrospective data regarding the rate of keratitis following lamellar surgeries are still limited.^9^

There are few publications related to surgical treatments used in PMD and the present case is the first report of keratitis after CLWR. Our patient exhibited infection in the late postoperative period. He had predisposing risk factors such as a loose suture and low socioeconomic level. Despite a good initial response to topical and systemic antifungal therapy, the patient was later treated with anterior chamber injections of antifungal drug because the infection penetrated to the deeper layers. The infectious episodes accompanied by recurrent hypopyon were attributed to anterior lens capsule injury and introduction of microorganisms to the lens during antifungal administration to the anterior chamber. After the infection was controlled, lensectomy was performed while showing extreme care to protect the posterior capsule barrier to prevent spread of infection to the vitreous and the intraocular lens was not implanted in the same session due to the possibility of microorganisms remaining in the capsular bag. Intraocular lens implantation was performed four months after lensectomy, when there was no further recurrence of infection and the fungus was believed to be eradicated. Finally, two months later, corneal cross-linking treatment was done both for antimicrobial purposes and to reinforce the resection area. After an extended follow-up period, both patients had good visual acuity without undergoing keratoplasty.

Although CLWR is effective and reliable for the treatment of PMD and less invasive than full-thickness techniques, unexpected complications may occur at each stage of treatment due to various factors. Treating these complications patiently and appropriately is important to achieve good visual outcomes.
